# Genome-Wide Analysis of miRNA Signature in the APPswe/PS1ΔE9 Mouse Model of Alzheimer's Disease

**DOI:** 10.1371/journal.pone.0101725

**Published:** 2014-08-22

**Authors:** Hongxue Luo, Qi Wu, Xiaoyang Ye, Yi Xiong, Jinyong Zhu, Junyu Xu, Yarui Diao, Duo Zhang, Maosheng Wang, Jinhua Qiu, Jianting Miao, Wei Zhang, Jun Wan

**Affiliations:** 1 Shenzhen Key Laboratory for Neuronal Structural Biology, Biomedical Research Institute, Shenzhen Peking University - The Hong Kong University of Science and Technology Medical Center, Shenzhen, Guangdong Province, China; 2 Ludwig Institute for Cancer Research, La Jolla, California, United States of America; 3 Department of Molecular, Cell and Developmental Biology, University of California Los Angeles, Los Angeles, California, United States of America; 4 GaoZhou people's Hospital, Gao Zhou, Guangdong Province, China; 5 Department of Neurology, Tangdu Hospital, Fourth Military Medical University, Xi'an City, Shanxi Province, China; 6 Division of Life Science, The Hong Kong University of Science and Technology, Clear Water Bay, Hong Kong, China; East Carolina University, United States of America

## Abstract

Alzheimer's disease (AD) is the most common cause of dementia. One of the pathological hallmarks of AD is amyloid β (Aβ) deposition. MicroRNAs (miRNAs) are small non-coding RNAs whose expression levels change significantly during neuronal pathogenesis and may be used as diagnostic markers. Some miRNAs are important in AD development by targeting genes responsible for Aβ metabolism. However, a systematic assessment of the miRNA expression profile induced by Aβ-mediated neuronal pathogenesis is still lacking. In the present study, we examined miRNA expression profile by using the APPswe/PS1**Δ**E9 mouse model of AD. Two sibling pairs of mice were examined, showing 30 and 24 miRNAs with significantly altered expression levels from each paired control, respectively. Nine known miRNAs were common in both groups. Prediction of putative target genes and functional annotation implied that these altered miRNAs affect many target genes mainly involved in PI3K/Akt signaling pathway. This study provides a general profile of miRNAs regulated by Aβ-associated signal pathways, which is helpful to understand the mechanism of Aβ-induced neuronal dysfunction in AD development.

## Introduction

Alzheimer's disease (AD) is common among elderly people and results in dementia that significantly affects the quelity of life in patients. The disease is characterized histologically by the appearance of senile plaque (SP) and neurofibrillary tangles (NFT). SP is caused by the accumulation of amyloid β (Aβ) peptide derived from the sequential cleavage of amyloid precursor protein (APP) by beta-site APP-cleaving enzyme 1 (BACE1) and the γ-secretase complex. Accumulating evidence indicates that microRNAs (miRNAs) regulate Aβ production, NFT formation, and neurodegeneration by targeting different genes [1]–[3].

MiRNAs are small non-coding RNAs which mainly bind to 3′ untranslated region (3′ UTR) of target mRNAs and regulate gene expression [4]. Thus far more than 2000 human miRNAs were discovered, targeting over 60% of human protein-encoding genes that are essential for cellular activities [5]. MiRNAs are abundantly expressed in the nervous system and play essential roles in the differentiation and function of neurons and glial cells, with plausible contribution to the pathogenesis of neurodegenerative diseases [6], [7]. Many recent studies found aberrant miRNA expression in AD brains, and proposed that deregulation of miRNA expression plays a central role in AD pathogenesis [7]. Most of these studies focused on the role of miRNA in APP and Aβ metabolism. For example, Wang et al found that the level of miRNA-107 is significantly lower in AD patients. BACE1 was identified as a target of miRNA-107, connecting the level of miRNA-107 to Aβ formation and neuronal pathogenesis [3]. Study from Herbert et al showed that expression levels of miRNA-29a/b-1 cluster are also decreased in the cortexes of sporadic AD patients, associated with a two- to five- fold increase in the level of BACE1 protein [1]. Other examples of the roles of miRNA in AD include the finding that MiR-124 regulates the APP mRNA alternative splicing. MiR-101, -520c, -147, -16, -20a, -644 and -153 were also reported to target 3′UTR of APP mRNA. MiR-107, -29a/b1/c, -9, -328 and -298 regulate the expression of BACE1. These miRNAs form a network that indirectly regulates the APP processing, Aβ production and accumulation [8].

However, alterations in miRNAs expression are not necessarily restricted to one pathogenic or metabolic chain. The up/down-regulation of certain miRNAs could stem from the changed nervous environment in AD, instead of being the reason for such change. In this study, we examined the miRNA profile in a mouse model of AD carrying APPswe and PS1**Δ**E9 transgenes. The APPswe/PS1**Δ**E9 double transgenic mice (hereby denoted as AD mice) express a chimeric mouse/human amyloid precursor protein (Mo/HuAPP695swe) and a mutant human presenilin 1 (PS1-**Δ**E9), both directed to CNS neurons. Both mutations are associated with early-onset AD in human. Senile plaques could be detected as early as 4 months of age in the brains of AD mice [9]. Phillips et al found that 6-month-old AD mice exhibited slower visuospatial learning than controls. In the visuospatial re-learning test performed at 9, 11, 13, 15, and 18 months of age, AD mice exhibited a decrease in the speed of re-learning the task compared to controls [10]. Other behavior tests including morris water maze experiments also showed that AD mice have impaired ability in spatial learning [11], [12]. Our study in the profile of miRNA expression in brains of AD mice showed that multiple miRNAs were up/down-regulated in the process of neurodegeneration. These changes may be involved in the pathogenesis of AD and could be used as early diagnostic markers for the disease in human.

## Materials and Methods

### Ethics statement

This study was performed in strict accordance with animal use protocols approved by the Committee for the Ethics of Animal Experiments, Shenzhen Peking University The Hong Kong University of Science and Technology Medical Center (SPHMC) (protocol number 2011-004). All animals were handled in accordance with the guidelines of the Committee for the Ethics of Animal Experiments, SPHMC. All efforts were made to minimize suffering.

### Animal preparation and extraction of tissue

APP695 with Swedish mutation K595N/M596L and PS1ΔE9 double-transgenic mice (APPswe/PS**Δ**E9) were purchased from the Model Animal Research Center of Nanjing University, with the original source of The Jackson Laboratory [13]. Two sibling pairs of wild-type (WT) and transgenic mice (WT1 vs APP4; WT2 vs APP3) of nine-month old were selected. The mice were anesthetized with pentobarbital (50 mg/kg) followed by the removing and dissection of the brain tissues. The cortexes of the brains were frozen in liquid nitrogen for the further RNA extraction.

### RNA extraction

The extraction and purification of total RNA, including small RNAs used for deep sequencing was carried out by using miRNeasy Mini Kit (Qiagen, US) according to the manufacture. The RNA for qRT-PCR was extracted from transgenic mice of different stages (n> = 3/type/age) by TRIZOL (Invitrogen, US) in accordance with the manufacture's protocol.

### Small RNAseq Library building and High-throughput sequencing

The RNAseq was performed by Shanghai Biotechnology Corporation. Briefly, total RNA qualified by Agilent Bioanalyzer 2100(Agilent technoloies Santa Clara,US)electrophoresis was then sequentially ligated with the RNA 3′ adapter (5′-pUCGUAUGCCGUCUUCUGCUUGidT-3′) and RNA 5′adapter (5′-GUUCAGAGUUCUACAGUCCGACGAUC-3′) using T4 RNA ligase. The RNA with bilateral adapters were reversely transcribed to cDNA by the RT primer (5′-CAAGCAGAAGACGGCATACGA-3′) for further PCR amplification. PCR amplification was performed with the primer set (5′-CAAGCAGAAGACGGCATACGA-3; 5′-AATGATACGGCGACCACCGACAGGTTCAGAGTTCTACAGTCCGA-3′) which annealed to the ends of the adapters. The PCR products needed to be size-fractionated on a 6% Novex TBE PAGE gel that differed from the two-step gel separation of small RNA before amplification discribed before [14]. After purification and quantification using Qubit dsDNA HS (Qubit 2.0 Fluorometer) and High Sensitivity DNA Chip (Aglient 2100), the recycled PCR products were used for Cluster Generation and sequencing on Illummina HiSeq 2000 in accordance with cBot and the HiSeq 2000 user guide. The raw sequencing data was uploaded to NCBI's Gene Expression Omnibus (GEO) which is accessible through GEO Series accession number GSE55589.

### Small RNAseq analysis process

Preliminary quality control of raw reads was carried out as the following programs, including discarding adaptor-adaptor contaminants and removing the low quality reads, such as reads that were shorter than 18 nucleotides and those overwhelming 35 nucleotides after being trimmed, and those containing fuzzy bases or low quality bases (<10). The rest were further filtered using fastx quality filter (fastx_toolkit-0.0.13.2). The qualified reads which passed the filters above were aligned to the mouse genome for more bioinformatics analysis.

All the small RNAs tags that past filters were sent to match with miRbase19.0 (http://www.mirbase.org/) using Bowtie [15] together with the other ncRNA databases:GRCm38.68.ncrna (http://asia.ensembl.org/index.html), Rfam V10 (http://rfam.sanger.ac.uk/), Prina (http://www.ncbi.nlm.nih.gov/) to profile the annotated small RNAs and calculate counts of the unique tags using CLC genomics workbench 5.5 commence software with at most two mismatches and two bases changed at each ends. The CLC could recognize the residual ambiguous bases in low quality and discarded them. MiRbase allowed no mismatch. Sequences that can not be classified by any database above but could be aligned to genome were termed as unannotated ones. Align all the small RNAs tags to the miRbase19.0 to abtain the reads of mature miRNA, Mature miRNA and variant miRNA with mutant and length changed. In view of the length of known miRNAs distributing around 21–23 nt, subsequently, only the length of small RNAs between 18–35 nt were retained for next gene mapping. Here, we mapped the length-limited sequences onto mouse genome (Mus_musculus.GRCm38.68.dna.toplevel.fa) to categorize and figure out those small RNAs tags with annotations. The remaining unannotated small RNAs were used for novel miRNA prediction.

The unannotated small RNA was deemed to be a novel miRNA candidate if it could mapped onto the mouse genome (Ensemble release-68). Further verifying step referred to “Minimum Free Energy”and “Randfold p-value”, related to the pre-miRNA second hairpin structure which could be predicted using RNAfold (http://rna.tbi.univie.ac.at/cgi-bin/RNAfold.cgi). All the process could also be finished by “miRCat” tool (contained in sRNA Toolkit).

### Real-time PCR

The quantification of the known and novel miRNAs were carried out using SYBR Green-based real-time PCR. Reverse transcription was performed with RevertAid First Strand cDNA synthesis Kit (Thermo, US) containing 100 ng template RNA, 4 µl of 5×Reaction buffer, 1 µl of Ribolock RNAse inhibitor (20 U/µl), 2 µl of 10 mM dNTP Mix and revertAid M-MuLV reverse transcriptase (200 U/µl) in each reaction (20 µl). The RT reaction was conducted in accordance to the protocol. The PCR reaction contained 0.4 µl of diluted cDNA, 5 µl 2×iQTM SYBR Green supermix (BioRad), 0.2 µl of 10× miScript universal primer and 1 ul of 10× miScript primers. Amplification was performed using CFX96 Real-Time PCR detection system (BioRad) as follows: 95°C for 5 min, followed by 40 cycles at 95°C for 10 s, 60°C for 20 s and 72°C for 20 s. Each RT- PCR reaction was performed in triplicate. Relative expression was calculated using the comparative Ct method while the U6 snRNA was used as the housekeeping gene.

### Detection of differential expressed miRNAs and prediction of target genes

Counts of every differently expressed miRNA in four samples were normalized to the total number of reads of all miRNA. Due to the small sample size, P-value based on fisher's exact test was inferred as the statistical significance. Meanwhile, False discovery rate (FDR) was calculated as a multiple hypothesis testing to control P-value. A special miRNA was considered to be significantly differently expressed if the P-value and FDR given by two methods were <0.05 in common and there was at least 1.5-fold change in normalized expressed level (the novel miRNA was set up to 2-fold).

The target genes for every differerntly expressed miRNA were predicted using miRDB (http://mirdb.org/miRDB/) [16], miRanda (http://www.microrna.org/microrna/home.do) [17] and targetScan (http://www.targetscan.org/) [18]. The targets of novel miRNAs were mainly analyzed by miRanda. DIAND-microT-CDS (v5) (http://diana.imis.athena-innovation.gr/DianaTools/index.php?r=microT_CDS/index) [19], [20] was also used to predict the target genes and incorporate different miRNAs into pathways annotated in KEGG. DIVID gene annotation tool (http://david.abcc.ncifcrf.gov/tools.jsp) was used for the annotation of KEGG pathways of genes and the gene Ontology (GO) terms [21], [22].

## Results

### Overview of sequencing data

**Figure 1:**
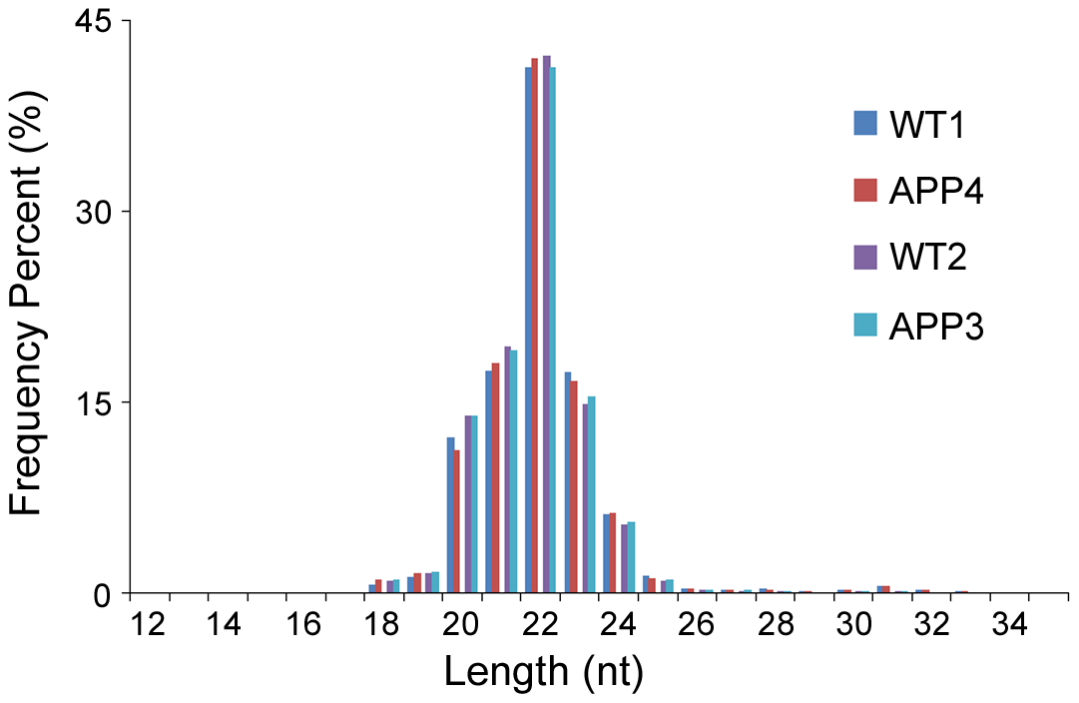
Length distribution of the annotated sequences. All four libraries from the two groups (WT1/APP4 and WT2/APP3) accumulate 22-nucleotide small RNAs, which is consistent with the typical size of miRNAs.

Illumina high throughput sequencing technology was employed in our study. After discarding the low quality reads and removing the reads with linkers, we obtained a total of 31265701, 24904512, 24281605 and 22851004 clean sequence reads which correspond to 580856 (WT1), 424443 (WT2), 492851 (APP3), 502564 (APP4) unique tags from the raw reads. The adequate saturation for each sample and the approximate reads between the two groups (WT and APP) allowed comparison in miRNA expression profile and distribution in each library. Further analysis showed that 95.9% (WT1), 96.7% (WT2), 96.2% (APP3) and 95.7% (APP4) of the clean reads can be annotated through matching four well-characterized small RNA databases: miRbase (mus muscumus), mus_muscumus.GRCm38.68.ncrna, Prina and Rfam V10 (Table S1). Annotated small RNAs showed a normal distribution pattern with the peak size located at 22 nt, in agreement with other studies in mammals (Figure 1).

### Categorization and mapping annotation

**Figure 2:**
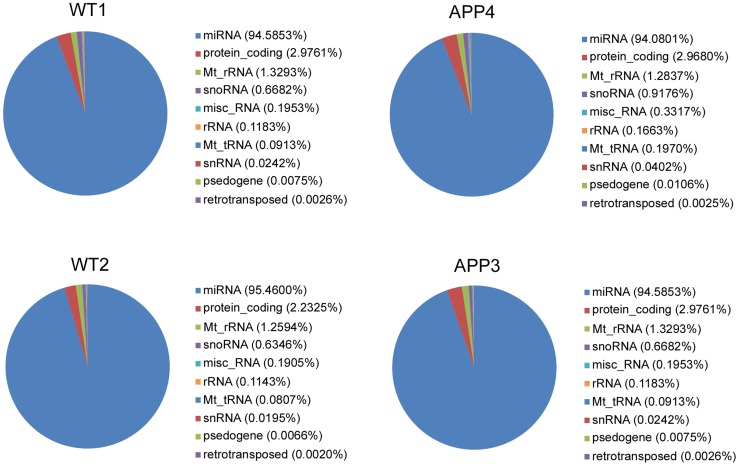
Annotation and distribution of small RNAs among different categories. snoRNA, small nucleolar RNA; mt, mitochondrial; rRNA, ribosobal RNA; tRNA, transfer RNA; snRNA, small nuclear RNA.

RNAs of 18–35 nt in length from the four libraries were aligned to the mouse genome (Mus_musculus.GRCm38.68.dna.toplevel.fa). We categorized small RNAs into known miRNA, protein_coding RNA, Mt_rRNA, snoRNA, misc_RNA, rRNA, Mt_tRNA, snRNA, pseudogene, and retro transposed RNA. More than 90% of the reads with known gene annotations are mapped to miRNAs. The second largest proportion can be mapped to protein coding genes, likely representing mRNA degradation products (Figure 2).

### MiRNAs are differentially expressed in mouse brains with AD

**Figure 3:**
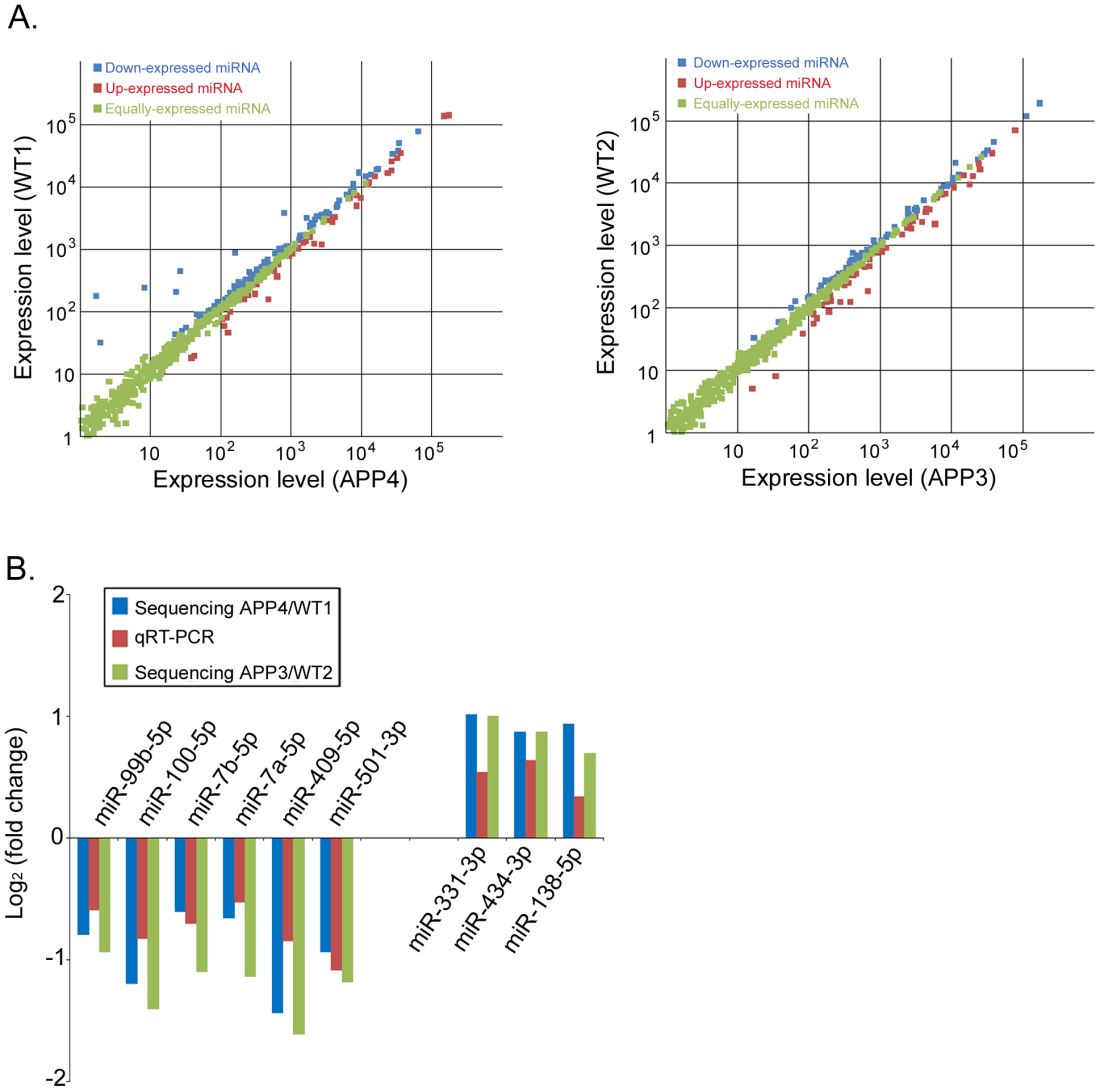
MiRNA expression levels from high throughput sequencing and verification with qRT-PCR. (A) The scatter plots show the miRNA expression levels of known miRNAs in group one (APP4/WT1) (Left) and group two (APP3/WT2) (Right). Green dots: equally expressed miRNAs between APP and WT; Blue dots: up-expressed miRNAs in APP sample compared with WT sample (p<0.05, q<0.05); Red dots: down-expressed miRNAs in APP sample compared with WT sample (p<0.05, q<0.05). (B) Selected miRNAs in both groups were verified by qRT-PCR.

Our sequencing data showed that 150 and 122 miRNAs (in group one and group two respectively. p<0.05 for both groups) are differentially expressed in AD brain compared to control (Figure 3A). MiRNAs that showed more than 1.5 fold change in their expression levels were shown in Table 1, listing 30 and 24 miRNAs from group one and two, respectively. Among them, nine miRNAs (miR-99b-5p, miR-7b-5p, miR-7a-5p, miR-501-3p, miR-434-3p, miR-409-5p, miR-331-3p, miR-138-5p and miR-100-5p) showed consistent changes in both groups. All 45 differentially expressed miRNAs identified from both groups were further examined by quantitative RT-PCR assay in 16 mice at the age of 9 months (8 AD mice and 8 WT mice). As shown in Figure 3B, except for miR-138-5p, expression levels of 8 out of 9 miRNAs showed consistent alteration in qRT-PCR experiment compared with the Illumina deep sequencing data. In contrast among the miRNAs which were altered in one group only, only miR-183-5p and miR-342-3p showed consistent trend of altered expression by comparing qRT-PCR result with sequencing (Figure S1A). It is evident that variation in individual mouse pair derives more alterations than those common in a larger experimental group, such as the one we employed by qRT-PCR to confirm the changes found in sequencing. Owning to abnormal initial counts, three differentially expressed miRNAs (miR-141-3p, miR-200c-3p, miR-96-5p) were not taken into account for comparison. Overall, the changed expression profiles of miRNAs in mouse brains harboring a neuronal pathogenic process similar to human AD indicate that these miRNAs may play a role in the process of AD development. We also compared miRNA expression levels from all four animals by using one single criterion (expression fold change >1.5 and p<0.05). 24 miRNAs were identified using this comparison (Table S2), which were all included in the 45 miRNAs listed in Table 1. QRT-PCR validation of the sequencing data is shown in Figure S1B.

**Table 1:** The top differentially expressed miRNA in APP4/WT1 and APP3/WT2.

APP4/WT1				APP3/WT2			
miRNA ID	log_2_(fold change)*	up/down expression	*p*-value adjusted	miRNA ID	log_2_(fold change)*	up/down expression	*p*-value adjusted
miR-200c-3p	7.10	up	2.16E-07	miR-331-3p	1.00	up	3.76E-06
miR-141-3p	6.74	up	2.72E-51	miR-211-5p	0.97	up	3.65E-02
miR-200a-3p	4.90	up	5.34E-60	miR-434-3p	0.89	up	0
miR-429-3p	4.08	up	4.73E-100	miR-383-5p	0.89	up	5.62E-25
miR-96-5p	4.04	up	3.67E-08	miR-1983	0.82	up	7.50E-04
miR-200b-3p	3.20	up	3.16E-39	miR-1843b-3p	0.69	up	6.91E-07
miR-183-5p	2.49	up	1.34E-123	miR-138-5p	0.69	up	2.41E-79
miR-182-5p	2.25	up	0	miR-6540-5p	0.65	up	4.39E-02
miR-331-3p	1.01	up	1.12E-04	miR-671-3p	0.62	up	1.92E-05
miR-138-5p	0.93	up	3.93E-106	miR-673-5p	0.60	up	8.98E-05
miR-434-3p	0.87	up	0	miR-125a-5p	−0.59	down	0
miR-376b-5p	0.77	up	1.45E-11	miR-125b-5p	−0.62	down	0
miR-423-3p	0.74	up	4.54E-07	miR-873a-3p	−0.66	down	4.67E-05
miR-15a-5p	0.74	up	5.61E-08	miR-146b-5p	−0.70	down	2.48E-78
miR-142-5p	0.73	up	3.62E-03	miR-185-5p	−0.93	down	1.83E-05
miR-451a	0.67	up	5.63E-04	miR-99b-5p	−0.93	down	0
miR-423-5p	0.64	up	1.19E-05	miR-221-5p	−1.04	down	6.32E-06
miR-543-3p	0.63	up	2.65E-03	miR-7b-5p	−1.10	down	8.40E-05
miR-29c-3p	0.59	up	6.01E-17	miR-7a-5p	−1.13	down	4.76E-10
miR-132-5p	0.59	up	1.31E-10	miR-501-3p	−1.18	down	5.69E-15
miR-7b-5p	-0.60	down	3.25E-03	miR-100-5p	−1.41	down	0
miR-7a-5p	-0.66	down	6.21E-07	miR-409-5p	−1.61	down	1.04E-30
miR-99a-5p	-0.77	down	4.59E-52	miR-342-3p	−1.87	down	1.23E-66
miR-99b-5p	-0.79	down	3.36E-218	miR-455-3p	−2.09	down	1.06E-04
miR-1249-3p	-0.80	down	9.52E-18				
miR-501-3p	-0.94	down	4.65E-05				
miR-5099	-1.11	down	3.69E-03				
miR-100-5p	-1.20	down	2.80E-138				
miR-409-5p	-1.44	down	7.33E-10				
miR-10b-5p	-1.61	down	5.05E-39				

*: fold change = APP/WT.

**Figure 4:**
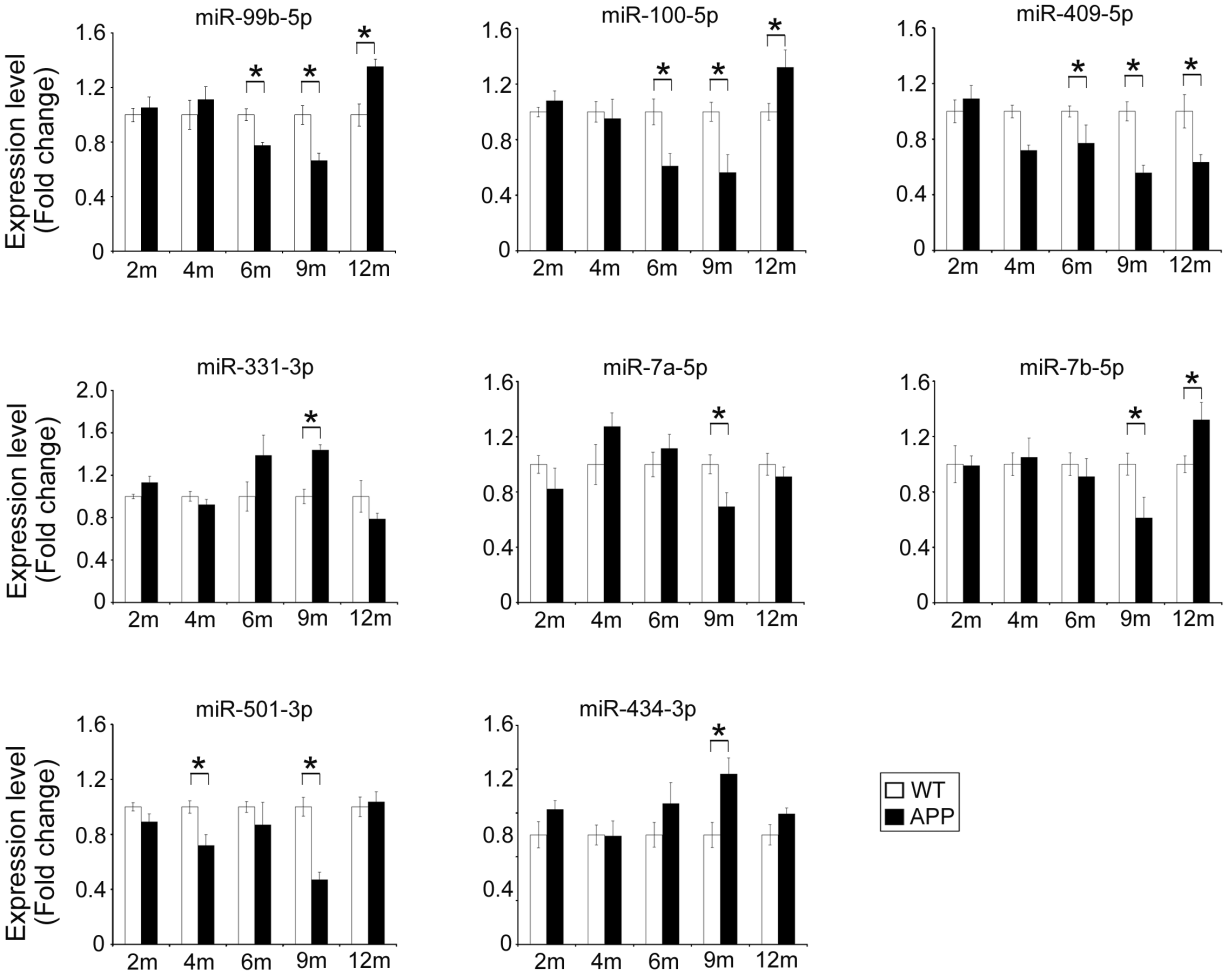
MiRNAs expression levels examined by qRT-PCR along the mice development. Six APPswe/PS1ΔE9 mice and six WT mice at each stage of 2, 4, 6 and 12 months of age, while 8 APPswe/PS1ΔE9 and 8 WT mice at 9 months old including the four mice for sequencing were examined.

Development of AD mandates a lengthy process where progression in neuronal pathogenesis accompanies with alteration of gene expression in different stages of the disease. To explore the temporal dynamic of miRNA expression change in AD,we examined the levels of miRNAs in AD mice with ages spanning from 2 to 12 months (Figure 4). Expression levels of miR-99b-5p and miR-100-5p were reduced in 6- and 9-month-old AD mice, but increased at 12 months of age. The level of miR-409-5p was lower since 6 months of age in AD brains. The other 5 miRNAs all showed significant alterations in expression levels at 9 months of age, but these alterations became either not significant (miR-331-3p, miR-7a-5p, miR-501-3p and miR-434-3p) or reversed (miR-7b-5p) at 12 months of age.

### Prediction of miRNA target genes

To explore the functional link between altered miRNA expression and AD development, we predicted the potential targets of two representative miRNAs, miR-331-3p and miR-409-5p, which were the top-elevated and top-reduced miRNAs in both groups, by three prediction methods (miRDB, miRanda and TargetScan). MiR-331-3p and miR-409-5p were predicted to each target 1138 and 745 genes (that are predicted in all three methods), Further analysis using GO database showed that the top biological processes affected by the predicted target genes are phosphate metabolic process and regulation of transcription (GO:0006796 and GO:0045449).

To analyze the interaction between miRNAs and protein-coding genes in the process of AD development in a comprehensive way, we performed combinational analysis for the gene target prediction. The 45 miRNAs that were shown to be differentially expressed in the sequencing experiment were predicted to target 10834 genes based on the online web server DIAND-microT-CDS(v5). To narrow down the pool of the potential targets and pathways involved, the p-value threshold and the microT threshold were set to be 0.05 and 0.8 respectively. We then restricted the algorithm on genes that belong to relative KEGG pathways using DIAND-miRPath v2.0 (http://diana.imis.athena-innovation.gr/DianaTools/index.php?r=mirpath/index) [23]. In the end, 92 pathways were implicated in the regulatory network, with the most significant pathways shown in Table 2.

**Table 2:** The pathways annotated by KEGG through DIAND-miRPath v2.0.

KEGG pathway	p-value	#genes	#miRNAs
Axon guidance	1.95E-41	69	27
PI3K-Akt signaling pathway	5.60E-29	127	29
MAPK signaling pathway	4.86E-28	102	28
Focal adhesion	4.62E-27	82	26
ErbB signaling pathway	1.47E-24	43	27
Pathways in cancer	5.91E-23	124	31
Regulation of actin cytoskeleton	4.66E-21	84	26
Wnt signaling pathway	3.56E-20	65	29
Gap junction	8.36E-18	37	22
Ubiquitin mediated proteolysis	4.59E-16	57	26
mTOR signaling pathway	3.42E-13	29	27
Transcriptional misregulation in cancer	1.21E-12	63	26
Adherens junction	2.31E-12	36	26
Insulin signaling pathway	2.40E-11	50	27
Long-term depression	6.83E-08	26	22
Long-term potentiation	1.16E-07	26	21
VEGF signaling pathway	1.57E-07	25	20
Glioma	3.06E-05	27	24
Notch signaling pathway	3.63E-05	18	18
Apoptosis	1.78E-03	28	19
Calcium signaling pathway	5.97E-03	49	25
p53 signaling pathway	6.06E-03	22	15
Tight junction	1.37E-02	41	26

#genes show the number of genes involved in each pathway; #miRNAs show the number of miRNAs which could target the genes involved in the pathways.

**Figure 5:**
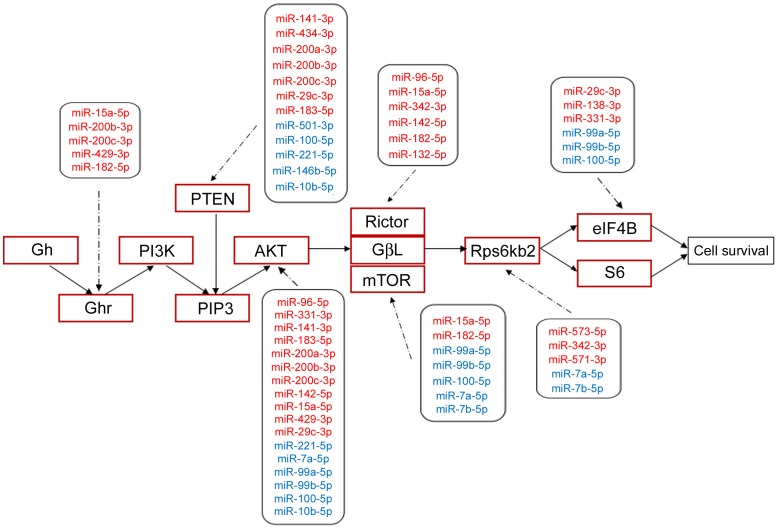
Differentially expressed miRNAs and their target genes in the PI3k/AKT signaling pathway. Joint analysis of KEGG and different algorithms was performed to identify the putative and experimentally validated targets for the most differently expressed miRNAs in two groups. Part of the pathway was displayed here. MiRNAs marked red are up-regulated while the blue ones are down-regulated.

The correlation between aberrantly expressed miRNAs and potential pathways was demonstrated in Figure S2. From the heatmap it is evident that different miRNAs play various roles in the potential pathways. It is worthwhile to mention that PI3K-Akt signaling pathway was simultaneously regulated by 29 miRNAs that showed aberrant expression in AD brains. 127 targeted genes are involved in PI3K/Akt signaling, implying an important role of PI3K/Akt in AD. Experimentally validated miRNA targets and part of the putative targets of differentially expressed miRNAs involved in this pathway are summarized in Figure 5.

## Discussion

MiRNAs are abundantly expressed in the brain and are essential for the functions of the neurons [24]. There are ample evidences showing that miRNAs regulate Aβ production and aggregation by targeting APP, BACE1 and other proteins involved in Aβ metabolism, and play important roles in the development of AD. In this study, we performed deep sequencing of miRNAs in the cortex of APPswe/PS1ΔE9 model of mouse AD. Aβ is significantly overexpressed in the barins of these mice, allowing the development of AD-like symptom in mice and subsequent characterization of miRNA profile described in this study. Among multiple miRNAs that were found to be dysregulated in AD brains, miR-409-5p showed constantly decreased expression level in AD brains from 4 months to 12 months of age, which is consistent with previous findings [25]. Although the target of miR-409-5p remained to be validated to provide further mechanistic insight, the early onset of its expression alteration suggests that miR-409-5p may play a role in the induction phase of AD, making it a potential target for early diagnosis and treatment of AD in huamn patients.

The level of miR-331-3p is elevated in the brains of 9-month-old AD mice compared to controls. Such alteration was not observed by comparing AD and WT mice at 4 months of age, but begins to emerge at 6 months of age when Aβ deposition starts, indicating that the dysregulation of miR-331-3p may stem from the accumulation of Aβ in AD brains. It's possible that miR-331-3p, by regulating the expression of its target genes, promotes the pathogenesis of AD in the context of increased Aβ level. It's note-worthy that the level of miR-331-3p becomes lower in AD brains at 12 months of age, possibly reflecting a negative-feedback mechanism linking the degree of AD disease with miR-331-3p expression level. In other systems, miR-331-3p was found to be up-regulated in acute and chronic lymphocytic leukemia and colorectal cancer [26], [27]. The expression of miR-331-3p may be regulated by genes involved in cell cycle control, such as E2F1, SOCS1, and genes involved in carcinogenesis and myogenesis [27]–[30]. It's possible that cellular changes in late-stage AD surpasses the accumulation of Aβ in regulating miR-331-3p expression level. Similar to miR-331-3p, some miRNAs such as miR-99b-5p, miR-100, miR-7b-5p and miR-501-3p also have a reversed expression change at late stage of AD (12 months) compared with those at relatively early AD stages (6 to 9 months). Some preliminay results from the experiments performed in neuronal cell lines indicate that the change of those miRNAs at early stages may protect neurons from Aβ-induced apoptosis (Data not shown). It suggests that some miRNAs change at early stage of AD may help to keep the cell homeostasis. Such interplay between Aβ accumulation, neuronal pathology and miRNA expression may further complicate the mechanism by which miRNAs regulate AD development, and remains to be future elucidated.

Each miRNA that showed differential expression levels in AD brains compared to controls can possibly target multiple genes. For example, miR-15a-5p may regulate Ghr, AKT, Rictor and mTOR, all of which related to protein synthesis and cell survival. On the other hand, a single gene may be regulated by more than one miRNA. PTEN, a classic tumor-suppressor gene, was shown to be targeted by at least 5 miRNAs (miR-141-3p, miR-200c-3p, miR-183-5p, miR-29c-3p and miR-221-5p) [31]–[34]. Therefore the interaction between miRNAs and proteins exhibits a network-like pattern and needs to be examined as a whole. Expression of a given protein depends on the dynamics of multiple miRNAs that are up- or down-regulated at specific stage of AD pathogenesis. PI3K/Akt/mTOR signaling is another pathway that is potentially regulated by miRNAs. Activation of PI3K/Akt signaling promotes the survival of neurons. MTOR functions downstream of PI3K/Akt and plays important roles in the process of cell growth, metabolism and aging [35], [36]. In the central nervous system, mTOR regulates neuronal differentiation, axon growth and navigation, dendritic arborization and synaptogenesis [37]. Previous reports showed that accumulation of Aβ interrupts PI3K/Akt/mTOR signaling [38], [39] while another study showed that neurons treated by Aβ oligomer exhibit elevated levels of activated Akt and mTOR [40]. Conversely, rapamycin, a mTOR inhibitor, could reduce Aβ level and ameliorate cognitive dysfunction in mouse model of AD [41]. As the activator of mTOR, PI3K/Akt signaling accelerates the extracellular glutamate clearance by regulating the function of GLT1 (glutamate transporter 1) in astrocytes, a process of AD [42]. Insufficient mTOR activity leads to neuron atrophy in the process of AD [43]. Several downstream targets of mTOR signaling, including 4E-BP1, eEF2, and eEF2 kinase, are also implicated in tau protein dynamics in AD brain [44], [45]. Changes in protein components of mTOR pathway, such as p70S6K, eIFα and PKR are also observed in AD [46]. The role of PI3K/Akt/mTOR signaling in synaptic plasticity, learning and memory formation were also illustrated by using rapamycin and genetically modified mice [47], [48]. Taken together, increasing evidence suggests that PI3K/Akt/mTOR may be a critical regulator of AD development. Our findings in the alterations of miRNA expression that potentially impact PI3K/Akt/mTOR signaling may provide further insight towards our understanding on the process of AD pathogenesis. Such alterations in miRNA levels may prove important in the diagnosis and/or targeted treatment of AD in human patients. One line of development can be the detection and profiling of miRNAs in the circulation that correlates to the development of AD. This can lead to more robust and invasion-free diagnostic methodology in complement with traditional methods involving collection of cerebrospinal fluid for the detection of tau protein.

## Supporting Information

Figure S1 **QRT-PCR verification of the miRNA expression levels.** (A) 30 and 24 miRNAs were found to have significantly change in the two groups respectively. Except the 9 miRNAs in both of the two groups and 3 with abnormal initial reads, all the miRNAs expression levels were verified by qRT-PCR in 8 APPswe/PS1ΔE9 and 8 WT mice at 9 months old of age (white bars). Sequencing results are shown as black bars. (B) Totally 24 miRNAs were found to have significantly change when comparing the data from all four animals but not pairly comparison. The qRT-PCR experiment was the same as that in (A).
(TIF)

Figure S2 **miRNAs versus pathways heat map (cluster based on significance levels).** Darker colors represent low significance values. The attached dendrograms on both axes depict hierarchical clustering results for miRNAs and pathways, respectively. On the miRNA axis, miRNAs clustered together were identified by exhibiting similar pathway targeting patterns. An analogous clustering can be observed on the pathway axis.
(TIF)

Table S1 **Small RNAs annotation.** A. Annotation of small RNAs reads matching with databases. B. Annotation of small RNAs with unique tags matching with databases. C. MiRNAs annotation according to miRBase v19.
(DOC)

Table S2 **The top differentially expressed miRNA in APP/WT.**
(DOCX)
