# Supplementary figures and images for: Genome-Wide Analysis of miRNA Signature in the APPswe/PS1ΔE9 Mouse Model of Alzheimer's Disease
Source: PLoS One. 2014 Aug 22;9(8):e101725. doi: 10.1371/journal.pone.0101725 (PMC4141691; doi:10.1371/journal.pone.0101725)

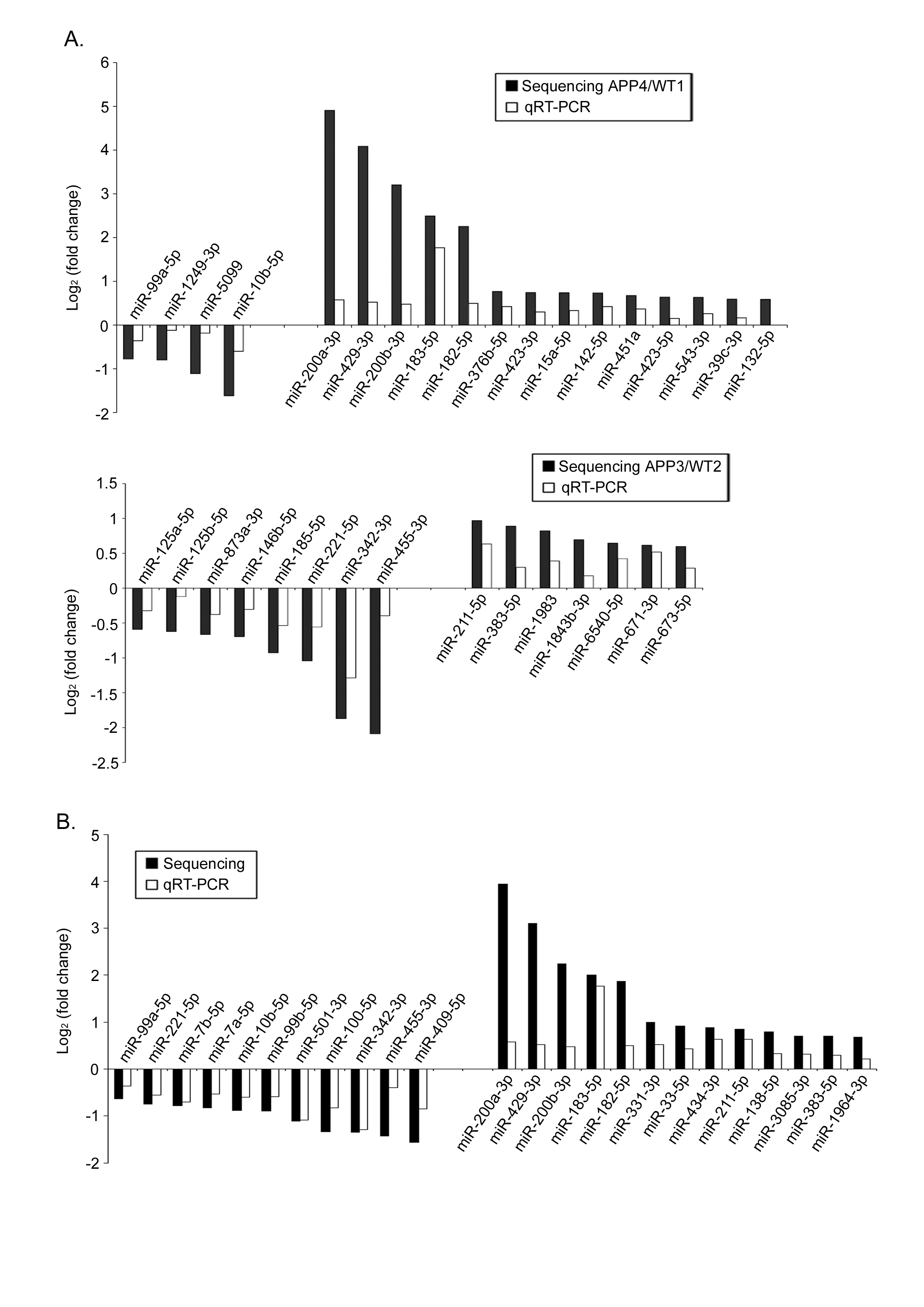

Supplement: Figure S1 — QRT-PCR verification of the miRNA expression levels. (A) 30 and 24 miRNAs were found to have significantly change in the two groups respectively. Except the 9 miRNAs in both of the two groups and 3 with abnormal initial reads, all the miRNAs expression levels were verified by qRT-PCR in 8 APPswe/PS1ΔE9 and 8 WT mice at 9 months old of age (white bars). Sequencing results are shown as black bars. (B) Totally 24 miRNAs were found to have significantly change when comparing the data from all four animals but not pairly comparison. The qRT-PCR experiment was the same as that in (A). (TIF) [file pone.0101725.s001.tif]

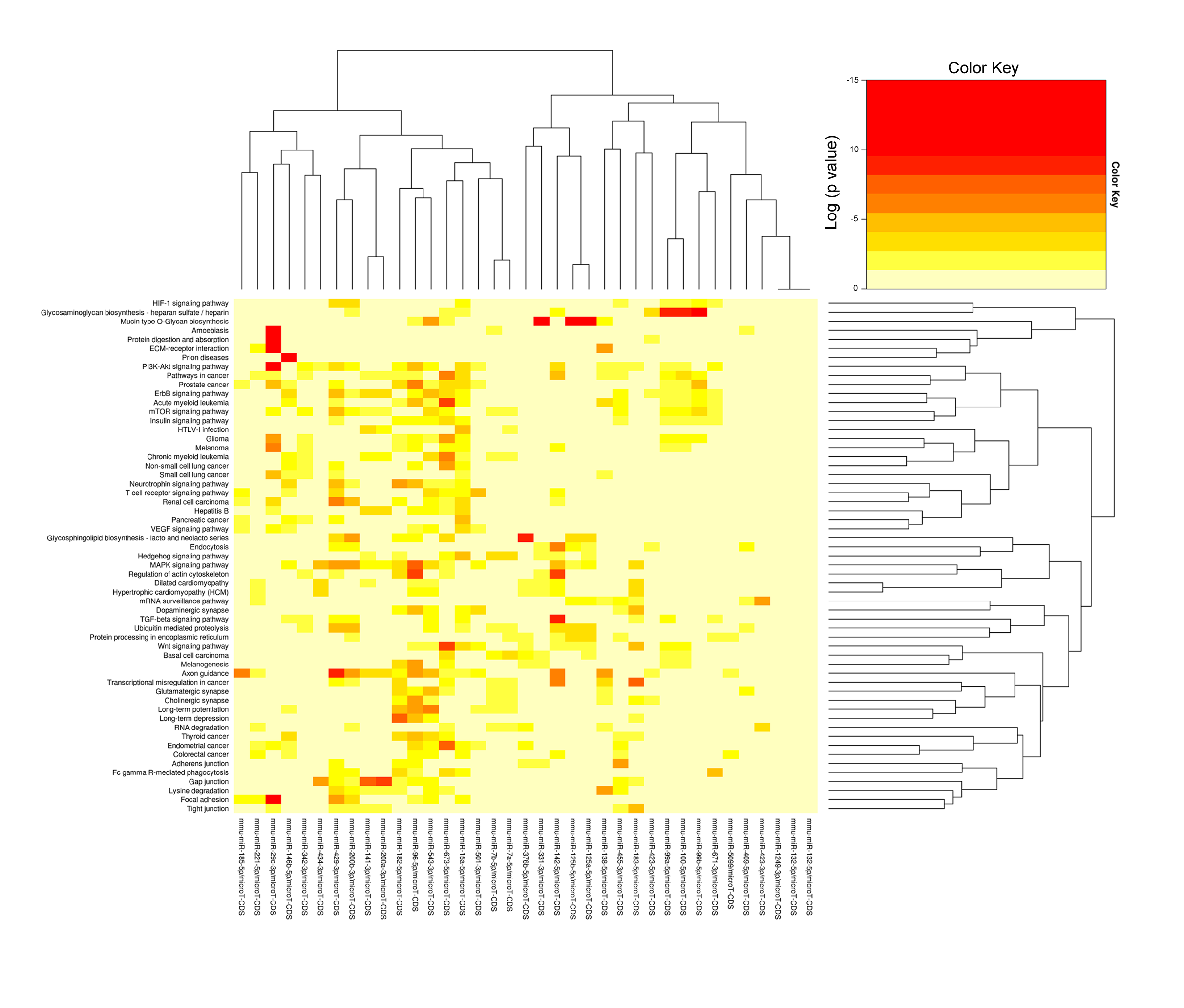

Supplement: Figure S2 — miRNAs versus pathways heat map (cluster based on significance levels). Darker colors represent low significance values. The attached dendrograms on both axes depict hierarchical clustering results for miRNAs and pathways, respectively. On the miRNA axis, miRNAs clustered together were identified by exhibiting similar pathway targeting patterns. An analogous clustering can be observed on the pathway axis. (TIF) [file pone.0101725.s002.tif]
