# Supplementary material for: Genome-Wide Analysis of miRNA Signature in the APPswe/PS1ΔE9 Mouse Model of Alzheimer's Disease
Source: PLoS One. 2014 Aug 22;9(8):e101725. doi: 10.1371/journal.pone.0101725 (PMC4141691; doi:10.1371/journal.pone.0101725)
Supplement: Table S1 — Small RNAs annotation. A. Annotation of small RNAs reads matching with databases. B. Annotation of small RNAs with unique tags matching with databases. C. MiRNAs annotation according to miRBase v19. (DOC) [file pone.0101725.s003.doc]

**Table S1. Small RNAs annotation.**

A. Annotation of small RNAs reads matching with databases.

|  | Counts (Percentage) | | | |  | Perfect matched (Percentage) | | | |
| --- | --- | --- | --- | --- | --- | --- | --- | --- | --- |
|  | WT1 | WT2 | APP3 | APP4 |  | WT1 | WT2 | APP3 | APP4 |
| annotated | 29972003 (95.90%) | 24094140 (96.70%) | 23366479 (96.20%) | 21878053 (95.70%) |  | 25668890 (85.60%) | 20767807 (86.20%) | 20101544 (86.00%) | 18827370 (86.10%) |
| with miRbase(mus muscumus) | 26936041 (89.90%) | 22745192 (94.40%) | 21925979 (93.80%) | 19813669 (90.60%) |  | 23421660 (87.00%) | 19807660 (87.10%) | 19081219 (87.00%) | 17360740 (87.60%) |
| with mus_muscumus. GRCm38.68.ncrna | 726616 (2.40%) | 466244 (1.90%) | 471187 (2.10%) | 522665 (2.40%) |  | 655138 (90.20%) | 431069 (92.50%) | 442181 (92.30%) | 471541 (90.20%) |
| with Prina | 387029 (1.30%) | 248690 (1.00%) | 275779 (1.20%) | 309163 (1.40%) |  | 97734 (25.30%) | 61935 (24.90%) | 70384 (25.50%) | 78045 (25.20%) |
| with Rfam V10 | 19922317 (6.40%) | 634014 (2.60%) | 685534 (2.90%) | 1232556 (5.60%) |  | 1494358 (77.70%) | 467143 (73.70%) | 507760 (74.10%) | 917044 (74.50%) |
| unannotated | 1293698 (4.10%) | 810372 (3.30%) | 915126 (3.80%) | 972951 (4.30%) |  |  |  |  |  |
| total | 31265701 (100.00%) | 24904512 (100.00%) | 24281605 (100.00%) | 22851004 (100.00%) |  |  |  |  |  |

|  | 1 mismatched (Percentage) | | | |  | 2 mismatched (Percentage) | | | |
| --- | --- | --- | --- | --- | --- | --- | --- | --- | --- |
|  | WT1 | WT2 | APP3 | APP4 |  | WT1 | WT2 | APP3 | APP4 |
| annotated | 3269084 (10.90%) | 2584591 (10.70%) | 2496063 (10.70%) | 2388204 (10.90%) |  | 1034029 (3.40%) | 741742 (3.10%) | 768872 (3.30%) | 662479 (3.00%) |
| with miRbase(mus muscumus) | 2667227 (9.90%) | 2281799 (10.00%) | 2186606 (9.90%) | 1939002 (9.80%) |  | 847154 (3.70%) | 655733 (2.90%) | 676154 (3.10%) | 514527 (2.60%) |
| with mus_muscumus. GRCm38.68.ncrna | 55721 (7.70%) | 28024 (6.00%) | 29207 (6.10%) | 40514 (7.80%) |  | 15757 (2.20%) | 7151 (1.50%) | 7799 (1.60%) | 10610 (2.00%) |
| with Prina | 258374 (66.80%) | 166568 (67.00%) | 182309 (66.10%) | 201458 (65.20%) |  | 30921 (8.00%) | 20187 (8.10%) | 23086 (8.40%) | 29660 (9.60%) |
| with Rfam V10 | 287762 (15.00%) | 108200 (17.10%) | 115941 (16.90%) | 207230 (16.80%) |  | 140197 (7.30%) | 58671 (9.30%) | 61833 (9.00%) | 107682 (8.70%) |

B. Annotation of small RNAs with unique tags matching with databases.

|  | WT1 | WT2 | APP3 | APP4 |
| --- | --- | --- | --- | --- |
| annotated | 147955 (25.50%) | 115231 (27.10%) | 120270 (24.40%) | 131023 (26.10%) |
| with miRbase(mus muscumus) | 63140 (42.70%) | 59094 (51.30%) | 59818 (49.70%) | 59205 (45.20%) |
| with mus_muscumus. GRCm38.68.ncrna | 31729 (21.40%) | 19313 (16.80%) | 21648 (18.00%) | 27198 (20.80%) |
| with Prina | 7318 (4.90%) | 5329 (4.60%) | 6197 (5.20%) | 6720 (5.10%) |
| with Rfam V10 | 45768 (30.90%) | 31495 (27.30%) | 32607 (27.70%) | 37900 (28.90%) |
| unannotated | 432901 (74.50%) | 309212 (72.90%) | 372581 (75.60%) | 371541 (73.90%) |
| total | 580856 (100.00%) | 424443 (100.00%) | 492851 (100.00%) | 502564 (100.00%) |

C. MiRNAs annotation according to miRBase v19.

|  | Total | Mature 5‘ total | Mature 5’ exactly matches | Mature 5‘ length variants | Mature 5’ mutant variants | Non-mature total | Mature 3‘ | Precursor |
| --- | --- | --- | --- | --- | --- | --- | --- | --- |
| WT1 | 26936041 | 15378844 | 7232618 | 6812901 | 1333325 | 11557197 | 10706924 | 850273 |
| WT2 | 22745192 | 12647943 | 5843393 | 5626839 | 1174720 | 10097249 | 9430769 | 666480 |
| APP3 | 21925979 | 11501362 | 5447127 | 5066250 | 996985 | 10414617 | 9756572 | 659045 |
| APP4 | 19813669 | 11278761 | 5567655 | 7432009 | 979097 | 8543908 | 7769983 | 764925 |
