# Supplementary material for: Genome-Wide Analysis of miRNA Signature in the APPswe/PS1ΔE9 Mouse Model of Alzheimer's Disease
Source: PLoS One. 2014 Aug 22;9(8):e101725. doi: 10.1371/journal.pone.0101725 (PMC4141691; doi:10.1371/journal.pone.0101725)
Supplement: Table S2 — The top differentially expressed miRNA in APP/WT. (DOCX) [file pone.0101725.s004.docx]

**Table S2. The top differentially expressed miRNA in APP/WT.**

|  | **Sequencing** | | |  | **qRT-PCR** |
| --- | --- | --- | --- | --- | --- |
| **miRNA ID** | **log_2_(fold change)*** | **up/down expression** | ***p*-value adjusted** |  | **log_2_(fold change)*** |
| mir-99a-5p | -0.63 | down | 0 |  | -0.36 |
| mir-221-5p | -0.74 | down | 1.44E-57 |  | -0.56 |
| mir-7b-5p | -0.78 | down | 6.96E-64 |  | -0.71 |
| mir-7a-5p | -0.82 | down | 3.24E-167 |  | -0.53 |
| mir-10b-5p | -0.88 | down | 5.70E-251 |  | -0.60 |
| mir-99b-5p | -0.89 | down | 0 |  | -0.59 |
| mir-501-3p | -1.11 | down | 1.30E-158 |  | -1.09 |
| mir-100-5p | -1.34 | down | 0 |  | -0.83 |
| mir-342-3p | -1.35 | down | 4.24E-06 |  | -1.29 |
| mir-455-3p | -1.42 | down | 6.81E-32 |  | -0.40 |
| mir-409-5p | -1.57 | down | 0 |  | -0.84 |
| mir-200a-3p | 3.94 | up | 0 |  | 0.58 |
| mir-429-3p | 3.10 | up | 0 |  | 0.53 |
| mir-200b-3p | 2.25 | up | 6.30E-268 |  | 0.48 |
| mir-183-5p | 2.00 | up | 0 |  | 1.77 |
| mir-182-5p | 1.87 | up | 0 |  | 0.50 |
| mir-331-3p | 1.00 | up | 1.75E-96 |  | 0.52 |
| mir-33-5p | 0.92 | up | 5.84E-07 |  | 0.43 |
| mir-434-3p | 0.88 | up | 0 |  | 0.64 |
| mir-211-5p | 0.85 | up | 4.50E-20 |  | 0.63 |
| mir-138-5p | 0.79 | up | 0 |  | 0.33 |
| mir-3085-3p | 0.71 | up | 1.24E-08 |  | 0.32 |
| mir-383-5p | 0.71 | up | 0 |  | 0.30 |
| mir-1964-3p | 0.68 | up | 6.38E-04 |  | 0.22 |

*: fold change = (APP3+APP4)/(WT1+WT2)
